# Supplementary material for: Adeno-associated virus serotype 9 antibody seroprevalence for patients in the United States with spinal muscular atrophy
Source: Mol Ther Methods Clin Dev. 2023 Sep 20;31:101117. doi: 10.1016/j.omtm.2023.101117 (PMC10562739; doi:10.1016/j.omtm.2023.101117)
Supplement: Document S1. Figures S1 and S2 and Table S1 [file mmc1.pdf]

OMTM, Volume *31*

Supplemental information

Adeno-associated virus serotype 9 antibody seroprevalence for patients in the United States with spinal muscular atrophy

John W. Day, Jerry R. Mendell, Arthur H.M. Burghes, Rudolf W. van Olden, Rishi R. Adhikary, and Keith W. Dilly

Table S1. Reference table for normalized titers

Lab	Technical results	Elevated/NE	Normalized titers
Athena	<1:25	NE	NE
Athena	1:25	Elevated	E+1
Athena	1:50	Elevated	E+2
Athena	1:100	Elevated	E+3
Athena	≥1:200	Elevated	E+4
CTL	<1:50	NE	NE
CTL	1:50	NE	NE
CTL	1:100	Elevated	E+1
CTL	1:200	Elevated	E+2
CTL	1:400	Elevated	E+3
CTL	1:800	Elevated	E+4
CTL	>1:800 & 1:1600	Elevated	E+4

CTL, Cellular Technology Limited; NE, not elevated. Note: For the Athena test, a titer concentration ≥1:25 was considered elevated, and for the CTL test, >1:50 was considered elevated.

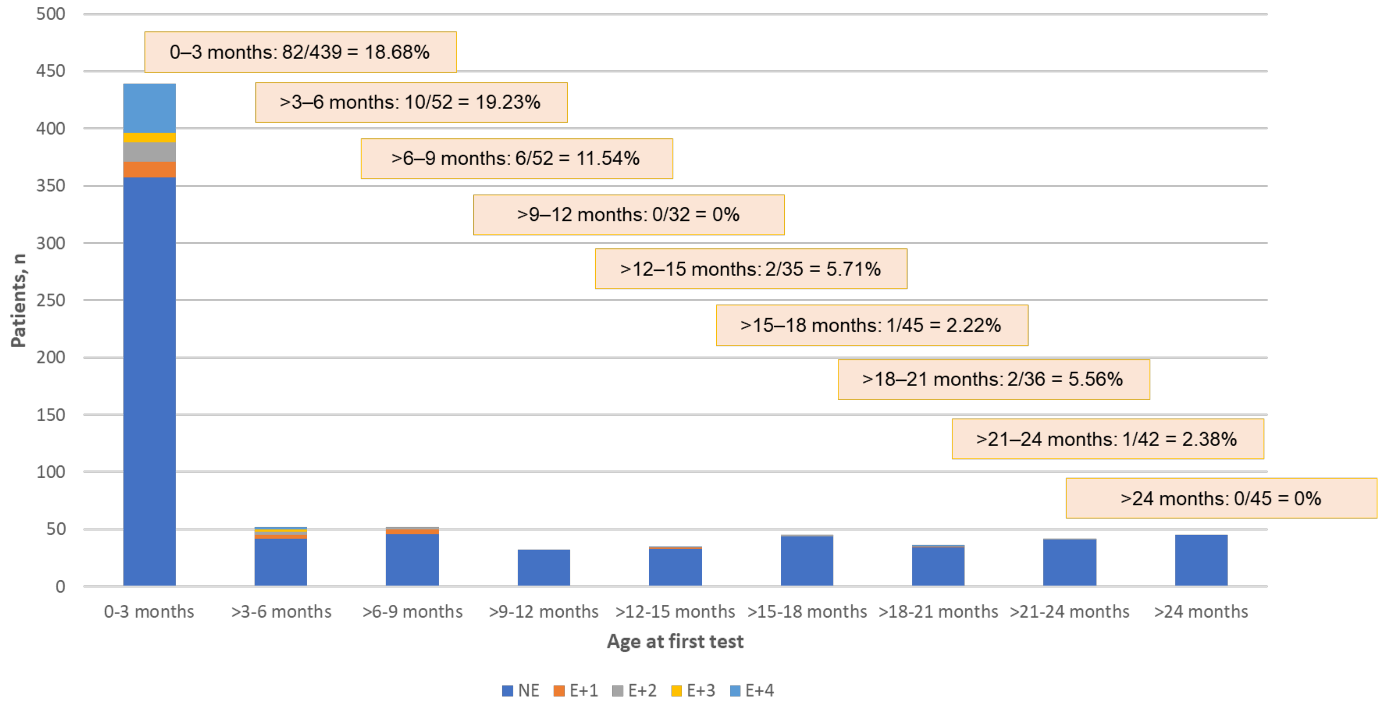

a) Patients with first test completed by Athena Diagnostics (n=778)

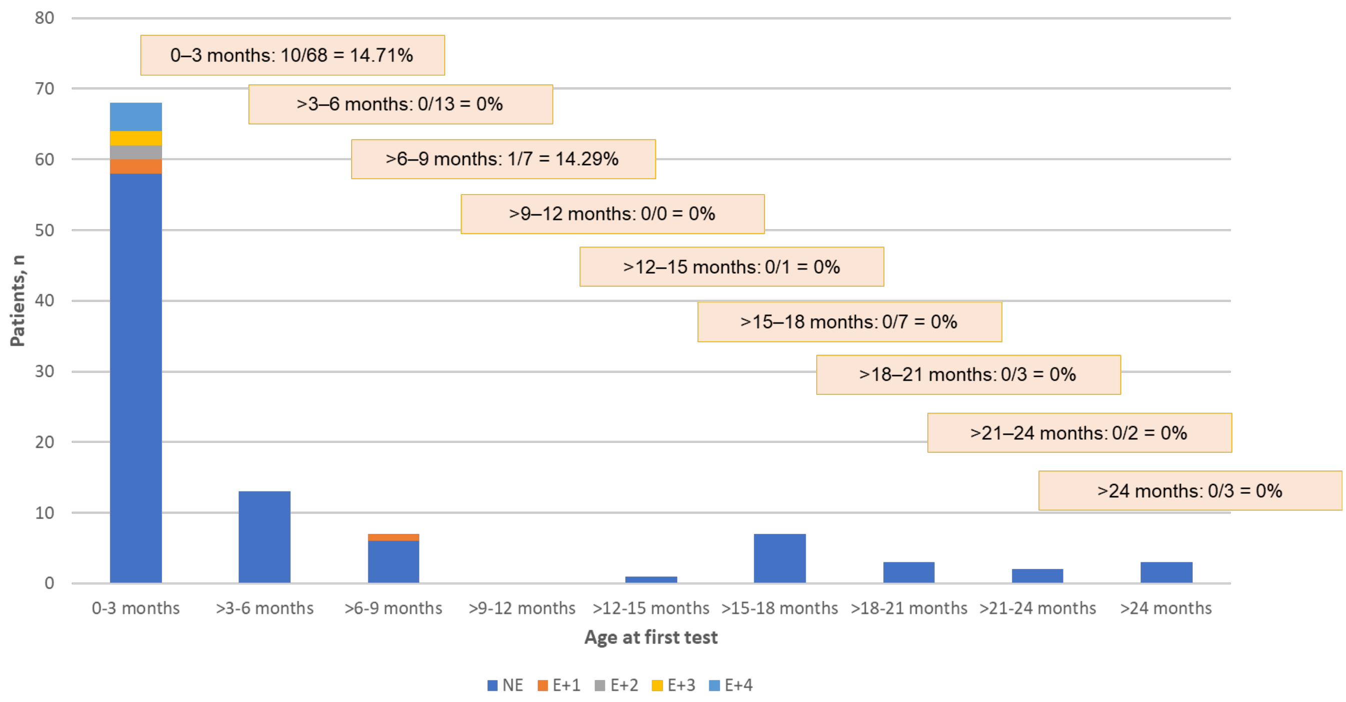

b) Patients with first test completed by CTL (n=104)

Figure S1. Age distribution of normalized titers at first test for antibodies that bind AAV9 according to laboratory that conducted the test.

CTL, Cellular Technology Limited; E+1, 1:25 titer concentration on Athena test or 1:100 titer concentration on CTL test; E+2, 1:50 titer concentration on Athena test or 1:200 titer concentration on CTL test; E+3, 1:100 titer concentration on Athena test or 1:400 titer concentration on CTL test; E+4, ≥1:200 titer concentration on Athena test or ≥1:800 titer concentration on CTL test; NE, not elevated. Note: For the Athena test, a titer concentration ≥1:25 was considered elevated, and for the CTL test, >1:50 was considered elevated.

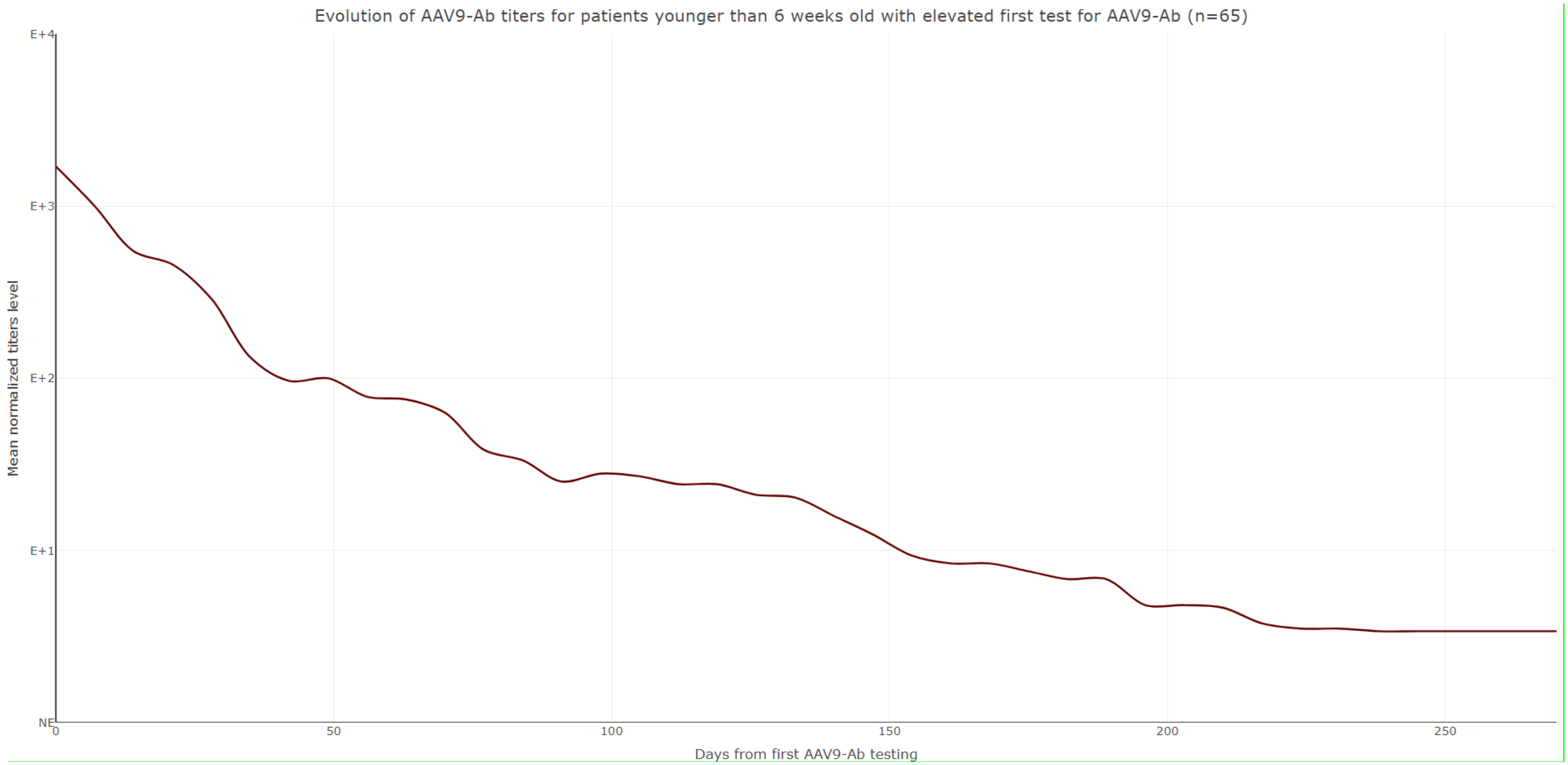

Figure S2. Evolution of AAV9-Ab titers for patients younger than 6 weeks old with elevated first test for AAV9-Ab who underwent repeat testing (n=65)

AAV9-Ab, adeno-associated virus serotype 9 antibodies.
