# Supplementary material for: Adeno-associated virus serotype 9 antibody seroprevalence for patients in the United States with spinal muscular atrophy
Source: Mol Ther Methods Clin Dev. 2023 Sep 20;31:101117. doi: 10.1016/j.omtm.2023.101117 (PMC10562739; doi:10.1016/j.omtm.2023.101117)
Supplement: Document S2. Article plus supplemental information [file mmc2.pdf]

Molecular Therapy
Methods & Clinical Development
Original Article

Adeno-associated virus serotype 9 antibody seroprevalence for patients in the United States with spinal muscular atrophy

John W. Day,[1] Jerry R. Mendell,[2,3] Arthur H.M. Burghes,[4] Rudolf W. van Olden,[5] Rishi R. Adhikary,[6] and Keith W. Dilly[7]

[1]Department of Neurology, Stanford University Medical Center, Stanford, CA, USA; [2]Center for Gene Therapy, Nationwide Children's Hospital, Columbus, OH, USA; [3]Department of Pediatrics and Department of Neurology, The Ohio State University, Columbus, OH, USA; [4]Department of Neurology and Department of Biological Chemistry and Pharmacology, The Ohio State University, Columbus, OH, USA; [5]Novartis Gene Therapies Switzerland GmbH, Rotkreuz, Switzerland; [6]CONEXTS-Real World Evidence, Novartis Healthcare Private Limited, Hyderabad, India; [7]Novartis Gene Therapies, Inc., Bannockburn, IL, USA

Onasemnogene abeparvovec is a recombinant adeno-associated virus serotype 9 (AAV9) vector-based gene therapy for spinal muscular atrophy (SMA). Patients with elevated titers of anti-AAV9 antibodies (AAV9-Ab) should not receive onasemnogene abeparvovec because of potential safety and efficacy implications. We conducted a retrospective study to describe the seroprevalence of anti-AAV9 binding antibodies for pediatric patients with SMA in the United States. At initial testing, 13.0% (115 of 882) of patients (mean [SD] age, 26.29 [33.66] weeks) had elevated AAV9-Ab titers. The prevalence of elevated titers decreased as age increased, with 18.2% (92 of 507) of patients ≤3 months old but only 1.1% (1 of 92) of patients ≥21 months old having elevated titers. This suggests transplacental maternal transfer of antibodies. No patterns of geographic variations in AAV9-Ab prevalence were confirmed. Elevated AAV9-Ab titers in children <6 weeks old decreased in all circumstances. Lower magnitudes of elevated titers declined more rapidly than greater magnitudes. Retesting was completed at the discretion of the treating clinician, so age at testing and time between tests varied. AAV9-Ab retesting should be considered when patients have elevated titers, and elevations at a young age are not a deterrent to eventual onasemnogene abeparvovec administration. Early disease-modifying treatment for SMA leads to optimal outcomes.

INTRODUCTION

Recombinant adeno-associated virus (AAV) vectors, particularly those of serotype 9 (AAV9), have been used in gene therapy to successfully transduce various cell types and cross the blood-brain barrier.[1–3] Spinal muscular atrophy (SMA) is one of the primary therapeutic applications for AAV9 vectors.[4–6]

SMA is a monogenic neuromuscular disease caused by a loss of or mutation in the *survival motor neuron 1* (*SMN1*) gene, which directs the production of most of the survival motor neuron (SMN) protein found in the body. A small amount of SMN protein is produced by the *survival motor neuron 2* (*SMN2*) genes, which are retained in SMA.[7] Complete absence of SMN protein is embryonically lethal, and low levels of SMN protein result in the motor neuron dysfunction and degeneration seen in SMA.[8,9] The presentation of SMA ranges from severe hypotonia and respiratory failure at birth to varying degrees of mobility impairment that affect respiration, feeding, communication, and the ability to sit, stand, and walk. Children with the most common presentation of SMA never develop the ability to sit unassisted and eventually require ventilatory support. Without disease-modifying treatment (DMT), these children rarely live past 2 years of age.[10,11]

Onasemnogene abeparvovec is an AAV9-based therapy that delivers a construct that contains SMN cDNA and expresses full-length human SMN mRNA. This delivery enables the ongoing production of a functional SMN protein.[12,13] This increased SMN protein expression leads to improved neuronal survival and motor function and increased survival for patients with SMA.[14–19]

Naturally occurring AAVs are not known to cause disease symptoms in humans, though exposure to wild-type AAVs does prompt an immune response, including production of neutralizing antibodies, which are a subset of total binding antibodies.[20,21] Newborn immunity is derived from placental transfer of antibodies that mothers have acquired during their lives owing to infection or viral exposure, including those to AAV. While these maternally derived antibodies provide passive immunity during the first few months of life, their concentrations decline over time.[22,23] Exposure to maternal AAV antibodies may also occur during breastfeeding, though the likelihood of antibodies entering infant circulation through the intestinal mucosa is low.[24] With a developed immune system, infants may also produce AAV antibodies in response to exposure to naturally occurring AAV serotypes.[25–27] Exposure to wild-type AAVs generally can occur early

Received 7 March 2023; accepted 14 September 2023;
https://doi.org/10.1016/j.omtm.2023.101117.

Correspondence: John W. Day, MD, PhD, Department of Neurology, Stanford University Medical Center, MC 5979, 213 Quarry Road, Stanford, CA 94304, USA.
E-mail: jwday@stanford.edu

This is an open access article under the CC BY license (http://creativecommons.org/licenses/by/4.0/).

Table 1. Demographics and clinical characteristics of first test for antibodies that bind AAV9

	Total, N (%)	Qualitative results of first test	
		Elevated first AAV9-Ab test, n (%)	Non-elevated first AAV9-Ab test, n (%)
**Patients**	882 (100)	115 (13.04)	767 (86.96)
Female	425 (48.19)	42 (4.76)	383 (43.42)
Male	457 (51.81)	73 (8.28)	384 (43.54)
Age at first test (wk)			
Mean (SD)	26.29 (33.66)	9.33 (16.95)	28.83 (34.79)
Median (range)	5.14 (0.14–163.86)	2.43 (0.29–93.00)	7.71 (0.14–163.86)
***SMN2* copies**	**Patients, n (%)**		
One	6 (0.68)	1 (0.11)	5 (0.57)
Two	434 (49.21)	63 (7.14)	371 (42.06)
Three	259 (29.37)	33 (3.74)	226 (25.62)
Four	22 (2.49)	2 (0.23)	20 (2.27)
More than four	43 (4.88)	7 (0.79)	36 (4.08)
Missing	118 (13.38)	9 (1.02)	109 (12.36)
**Laboratory for first AAV9-Ab test**	**Patients, n (%)**		
Athena	778 (88.21)	104 (11.79)	674 (76.42)
CTL	104 (11.79)	11 (1.25)	93 (10.54)
**Number of AAV9-Ab tests**	**Patients, n (%)**		
At least two tests	141 (15.99)	83 (9.41)	58 (6.58)
Two tests	80 (9.07)	30 (3.40)	50 (5.67)
Three tests	35 (3.97)	27 (3.06)	8 (0.91)
Four tests	8 (0.91)	6 (0.68)	2 (0.23)
Five or more tests	18 (2.04)	14 (1.59)	4 (0.45)

AAV9-Ab, adeno-associated virus serotype 9 antibody; CTL, Cellular Technology Limited; SD, standard deviation; *SMN2*, *survival motor neuron 2* gene.

in life and continue through adulthood. Correspondingly, the prevalence of antibodies to AAVs generally increases with age, though results vary markedly between studies and geographies.[25,28–31]

The viral capsids of wild-type AAVs and recombinant AAVs are identical.[20] Therefore, greater concentrations of circulating anti-AAV9 antibodies (AAV9-Ab) may have safety and efficacy implications in gene therapy,[4,32] including immune responses[2,20,33,34] and reduced transduction of cells.[35] Low circulating concentrations of antibodies against AAV capsids used for gene therapy could favor increased safety and efficacy.[2] As such, patients may not receive onasemnogene abeparvovec if they have elevated AAV9-Ab titers.[36] Indirect enzyme-linked immunosorbent assay (ELISA) tests detecting immunoglobulin G using a 3,3′,5,5′-tetramethylbenzidine readout are used to determine circulating concentrations of total antibodies that bind AAV9,[2] and the degree that is considered "elevated" is dependent on the vendor test used.

A detailed time course of the clearance of maternal placentally transferred antibodies is unclear, especially for patients with SMA. The aim of this study was to provide a better understanding of AAV9-Ab seroprevalence for pediatric patients with SMA.

RESULTS

Patient population

A total of 882 patients were included in the analysis (425 [48.2%] female; 457 [51.8%] male) (Table 1). The mean (standard deviation [SD]) age at the first test for AAV9-Ab was 26.29 (33.66) weeks (median [range], 5.14 [0.14–163.86] weeks).

Most patients (778 [88.2%]) underwent initial testing for antibodies that bind AAV9 using the Athena test, and the remaining (104 [11.8%]) underwent initial testing with the Cellular Technology Limited (CTL) test. A total of 141 patients (16.0%) underwent retesting: 83 (58.9%) had an elevated first test and 58 (41.1%) had a non-elevated first test. Of the patients who underwent retesting, 80 (9.1%) patients had two tests, 35 (4.0%) had three, eight (0.9%) had four, and 18 (2.0%) had five or more retests. For those patients who repeated testing, 72 of 141 (51.1%) underwent Athena testing and 69 of 141 (48.9%) underwent CTL testing by the final test.

Prevalence of elevated AAV9-Ab titers

Of all 882 patients, 115 (13.0%) had an elevated first test for antibodies that bind AAV9: 42 of the 425 (9.9%) female patients and 73 of the 457 (16.0%) male patients (Table 1). Patients with elevated

Table 2. Age distribution of normalized titers at first test for antibodies that bind AAV9

	All,N (%)	NE, n (%)	E+1, n (%)	E+2, n (%)	E+3, n (%)	E+4, n (%)
All ages, mo	882 (100.0)	767 (86.96)	26 (2.95)	26 (2.95)	12 (1.36)	51 (5.78)
0–1	414 (100.0)	340 (82.13)	11 (2.66)	12 (2.90)	10 (2.42)	41 (9.90)
>1–2	59 (100.0)	47 (79.66)	2 (3.39)	5 (8.47)	0 (0)	5 (8.47)
>2–3	34 (100.0)	28 (82.35)	3 (8.82)	2 (5.88)	0 (0)	1 (2.94)
>3–4	18 (100.0)	13 (72.22)	1 (5.56)	1 (5.56)	1 (5.56)	2 (11.11)
>4–5	20 (100.0)	17 (85.00)	1 (5.00)	2 (10.00)	0 (0)	0 (0)
>5–6	27 (100.0)	25 (92.59)	1 (3.70)	0 (0)	1 (3.70)	0 (0)
>6–7	19 (100.0)	15 (78.95)	3 (15.79)	1 (5.26)	0 (0)	0 (0)
>7–8	22 (100.0)	21 (95.45)	0 (0)	1 (4.55)	0 (0)	0 (0)
>8–9	18 (100.0)	16 (88.89)	2 (11.11)	0 (0)	0 (0)	0 (0)
>9–10	9 (100.0)	9 (100.0)	0 (0)	0 (0)	0 (0)	0 (0)
>10–11	9 (100.0)	9 (100.0)	0 (0)	0 (0)	0 (0)	0 (0)
>11–12	14 (100.0)	14 (100.0)	0 (0)	0 (0)	0 (0)	0 (0)
>12–13	10 (100.0)	9 (90.00)	1 (10.00)	0 (0)	0 (0)	0 (0)
>13–14	11 (100.0)	11 (100.0)	0 (0)	0 (0)	0 (0)	0 (0)
>14–15	15 (100.0)	14 (93.33)	0 (0)	0 (0)	0 (0)	1 (6.67)
>15–16	16 (100.0)	16 (100.0)	0 (0)	0 (0)	0 (0)	0 (0)
>16–17	19 (100.0)	19 (100.0)	0 (0)	0 (0)	0 (0)	0 (0)
>17–18	17 (100.0)	16 (94.12)	0 (0)	1 (5.88)	0 (0)	0 (0)
>18–19	10 (100.0)	9 (90.00)	1 (10.00)	0 (0)	0 (0)	0 (0)
>19–20	12 (100.0)	12 (100.0)	0 (0)	0 (0)	0 (0)	0 (0)
>20–21	17 (100.0)	16 (94.12)	0 (0)	0 (0)	0 (0)	1 (5.88)
>21–22	15 (100.0)	15 (100.0)	0 (0)	0 (0)	0 (0)	0 (0)
>22–23	12 (100.0)	12 (100.0)	0 (0)	0 (0)	0 (0)	0 (0)
>23–24	17 (100.0)	16 (94.12)	0 (0)	1 (5.88)	0 (0)	0 (0)
>24	48 (100.0)	48 (100.0)	0 (0)	0 (0)	0 (0)	0 (0)

AAV9, adeno-associated virus serotype 9; CTL, Cellular Technology Limited; E+1, 1:25 titer concentration on Athena test or 1:100 titer concentration on CTL test; E+2, 1:50 titer concentration on Athena test or 1:200 titer concentration on CTL test; E+3, 1:100 titer concentration on Athena test or 1:400 titer concentration on CTL test; E+4, ≥1:200 titer concentration on Athena test or ≥1:800 titer concentration on CTL test; NE, not elevated.
Note: For the Athena test, a titer concentration ≥1:25 was considered elevated, and for the CTL test, >1:50 was considered elevated.

titers were younger than those with non-elevated titers (Table 1), and the percentage of patients with elevated titers decreased as patient age at first test increased (Table 2; Figure 1). Prevalence was greatest for patients aged ≤3 months old (92 of 507; 18.2%) and decreased markedly after 9 months of age, consistent with a decline of passively transferred antibodies over time. In the youngest age groups, 74 of 414 (17.9%) patients aged <1 month, 12 of 59 (20.3%) patients aged >1–2 months, and 6 of 34 (17.6%) patients aged >2–3 months had elevated titers. A similar pattern of elevated titers was observed when patients were stratified by laboratory, with the prevalence of seropositivity greatest at <3 months old (Athena: 82 of 439 [18.68%], CTL: 10 of 68 [14.71%]) (Figure S1).

Geographic variations in AAV9-Ab seroprevalence

There are potential geographic variations in AAV9-Ab seroprevalence, but no clear patterns were discernable from this analysis (Figure 2). For all patients tested, Wyoming, Kentucky, Missouri, and North Carolina had the greatest percentage of patients with elevated titers (2 of 4 [50.0%], 6 of 17 [35.3%], 7 of 21 [33.3%], and 6 of 20 [30.0%], respectively). For patients aged <1 month at the time of testing, North Carolina, Kentucky, Missouri, and Oklahoma had the greatest percentages with elevated titers (4 of 7 [57.1%], 6 of 15 [40.0%], 6 of 16 [37.5%], and 2 of 6 [33.3%], respectively). For all patients, Wisconsin (n = 18), Massachusetts (n = 10), Oregon (n = 8), West Virginia (n = 7), New Hampshire (n = 6), and Connecticut (n = 4) had no patients with elevated titers. Pennsylvania (n = 16), Wisconsin (n = 14), Massachusetts (n = 6), Louisiana (n = 5), and New Jersey (n = 4) had no patients who were tested at <1 month old with elevated titers. Large variations in the sample sizes from each state exist, and states with three or fewer patients were excluded from this analysis.

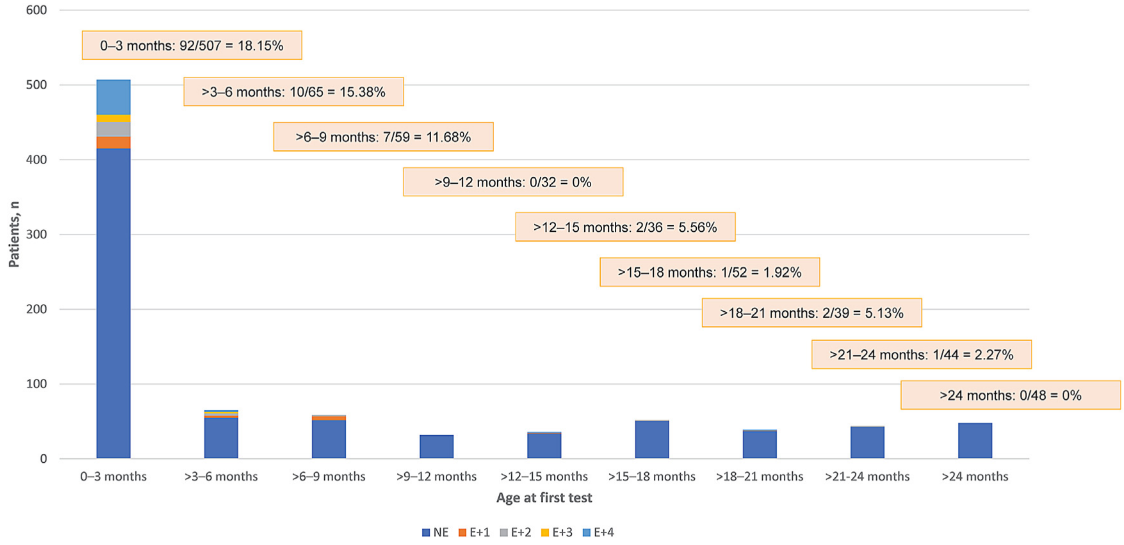

Figure 1. Age distribution of normalized titers at first test for antibodies that bind AAV9 (N = 882)

Prevalence greatest at ≤3 months old (18.2%) and decreased after 9 months of age (0%). CTL, Cellular Technology Limited; E+1, 1:25 titer concentration on Athena test or 1:100 titer concentration on CTL test; E+2, 1:50 titer concentration on Athena test or 1:200 titer concentration on CTL test; E+3, 1:100 titer concentration on Athena test or 1:400 titer concentration on CTL test; E+4, ≥1:200 titer concentration on Athena test or ≥1:800 titer concentration on CTL test; NE, not elevated. Note: For the Athena test, a titer concentration ≥1:25 was considered elevated, and for the CTL test, >1:50 was considered elevated.

Changes in AAV9-Ab seroprevalence

Antibody titers often became non-elevated when patients underwent repeat testing. Most patients had non-elevated titers with repeat tests over time. In all, 65 patients who were ≤6 weeks of age at the first test and had an elevated first test result underwent repeat testing (Figure 3). By the last test, all patients except one had seroconverted to non-elevated concentrations. Of note, the patient who did not seroconvert had elevated titers on eight repeat testings up to 7 weeks of age. No additional testing was completed until a final test at 14 months of age, at which time this ninth test still demonstrated elevated titers. No additional follow-up was conducted for this patient. A few patients (n = 4) temporarily seroconverted from non-elevated to elevated titers over the course of retesting, which was determined to be a result of undergoing testing at different laboratories. A small number of patients (n = 8) underwent retesting despite non-elevated test results early in the study period. These tests were conducted at the discretion of the treating clinician and may have been requested because of concerns about the length of time a non-elevated test was valid.

Time to test for AAV9-Ab seroprevalence

Health care providers retested for antibodies that bind AAV9 at varied intervals according to their discretion (Figure 4). For patients who underwent multiple AAV9-Ab tests (n = 141), the median time between tests ranged from 22.75 to 31.00 days. The shortest time between tests was for tests beyond the fourth testing, and the remaining intervals were nearly identical.

Time to non-elevated AAV9-Ab concentrations

For most patients, titers for antibodies that bind AAV9 decreased from elevated to non-elevated (Figures 3 and S2), and these patients were treated with onasemnogene abeparvovec according to guidelines directed by the US Food and Drug Administration (FDA). Most patients were able to receive treatment within a few weeks to months after the first elevated result as titers decreased to non-elevated concentrations with repeat tests. Lower magnitudes of elevated titers became non-elevated more rapidly than greater magnitudes of elevated titers (Figures 5A–5E). Patients with lower magnitudes of elevated titers reached non-elevated levels within 6 weeks, while patients with higher titers took up to several months to reach non-elevated titer levels.

DISCUSSION

The seroprevalence of elevated AAV9-Ab was low for infants with SMA. The percentage of infants with elevated titers was greatest in the neonatal and early infant periods and decreased over time. This was the result of passive transfer of antibodies from mother to child through the placenta, which has functional implications in extra-immunological early postnatal development. For most children in this study, anti-AAV9-Ab titers were low enough, either at initial testing or after repeat testing, to receive treatment with onasemnogene abeparvovec. The single patient with continually elevated titers is not consistent with our pathophysiologic understanding of maternal antibody transfer and waning of antibody titer levels over time in newborns. One possible explanation for the sustained elevation in AAV9-Ab titers was environmental exposure during the prolonged (>12 months) period without testing. While pivotal trials, managed access programs, and FDA-directed guidelines stipulate AAV9-Ab titers ≤1:50 to receive onasemnogene abeparvovec therapy based on limited human data, it remains unknown if preexisting antibodies impact the safety and efficacy of gene therapy in

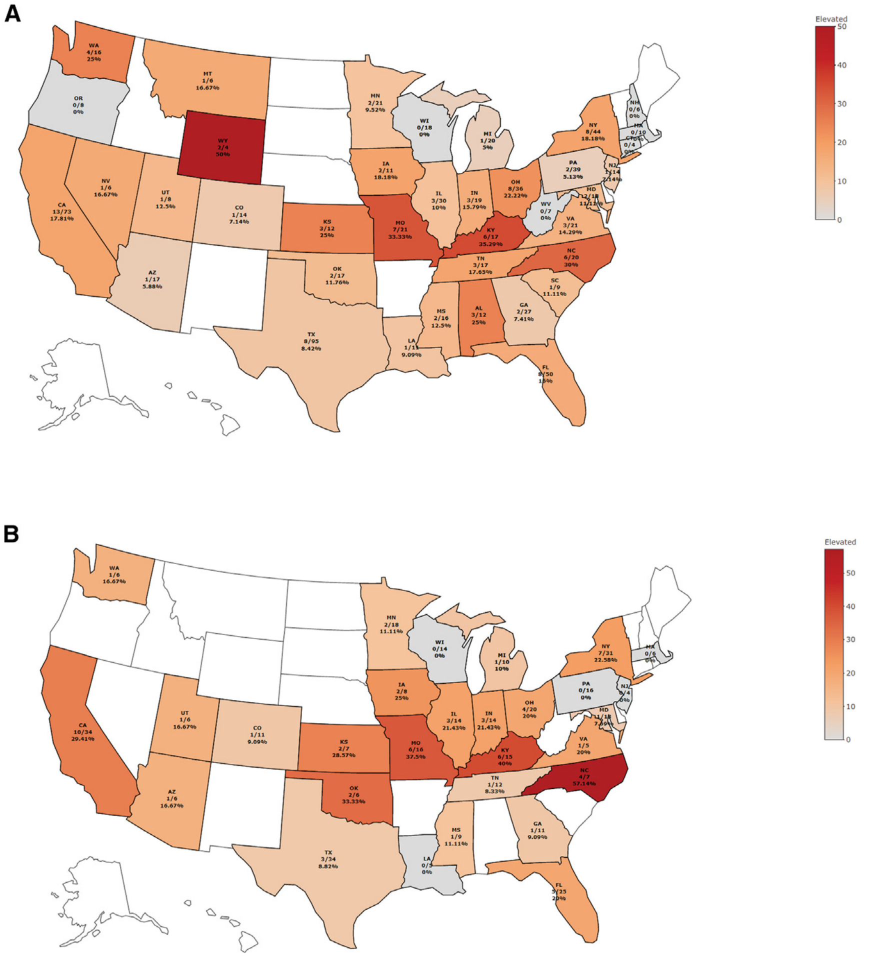

Figure 2. Potential regional variations in AAV9-Ab seroprevalence[a]

(A) Patients with elevated titers by state (n = 794).

(B) Patients <1 month old with elevated titers by state (n = 383).

AAV9-Ab, adeno-associated virus serotype 9 antibody.

[a]States with a small sample size (n ≤ 3) are not included.

humans. Of note, AAV antibody titers ≤1:400 for a different, but related, vector (AAV4h74) are acceptable for vector delivery in Duchenne muscular dystrophy,[37] which may have implications for gene therapy in SMA.

When onasemnogene abeparvovec treatment for SMA is a goal for health care providers and families, determining treatment appropriateness by testing and retesting AAV9-Ab concentrations as soon as possible is preferred. Repeat testing is recommended for patients with initially elevated titers and may be considered for those who have a prolonged period between initial antibody testing and onasemnogene abeparvovec dosing because of the risk of new exposure to environmental AAV9 and seroconversion.[38] When baseline titers are elevated, the retesting frequency is determined at the discretion of the treating physician but should not be a deterrent to eventual onasemnogene abeparvovec administration. As demonstrated with repeat testing, elevated titer concentrations may decrease within a few weeks, with lower magnitudes of elevated titers becoming non-elevated more rapidly than greater magnitudes of elevated titers, and treatment with onasemnogene abeparvovec may be able to proceed without a prolonged wait.

Early treatment for SMA is crucial for achieving optimal outcomes,[18,19] and SMA treatment should not be delayed, whether because of AAV9-Ab titers or other logistic or coverage concerns.[39–41] Bridge treatment with another DMT can be considered if patients are not yet candidates for onasemnogene abeparvovec treatment. In addition to onasemnogene abeparvovec, two other approved DMTs for SMA are available: nusinersen, an antisense oligonucleotide, and risdiplam, a small-molecule drug. These two treatments affect *SMN2* splicing, ultimately increasing the availability of functional SMN protein.[42] Nusinersen and risdiplam are not gene therapies, nor are they related to AAV9, so AAV9-Ab concentrations do not influence their safety or efficacy.

Although no consensus currently exists regarding combination or sequential use of DMTs,[43,44] there is anecdotal evidence that describes the use of nusinersen or risdiplam before onasemnogene abeparvovec administration,[45–48] and bridge treatments may be considered when onasemnogene abeparvovec administration is not possible owing to elevated AAV9-Ab titers or other factors.[49] However, clinical evidence for the rationale of ongoing combination therapy remains unclear, and more data are needed.[45–48]

Few data are available to describe the prevalence of AAV9-Ab for patients with SMA, but our results are similar to those of published studies. In a study of 34 patients (mean age 8.46 ± 7.10 months) in Israel screened for treatment with onasemnogene abeparvovec, six (17.65%) had elevated titers at initial testing. Of these six, three seroconverted to non-elevated titers upon repeat testing within 1.5–4.5 months after the initial test (at ages 2, 5, and 8 months).[50] Similarly, in our current analysis, 92 of 507 (18.2%) patients aged ≤3 months had elevated titers, and 115 of 882 (13.0%) total patients had elevated titers.

In a study of healthy children aged 2 to 7 years, the prevalence of AAV9-Ab was 6.0%.[29] In our study, for the oldest group of children tested (aged at least 21 to more than 24 months), 1.1% (1 of 92) had elevated titers. Finally, an analysis of infants screened for SMA treatment with onasemnogene abeparvovec in clinical trials and managed

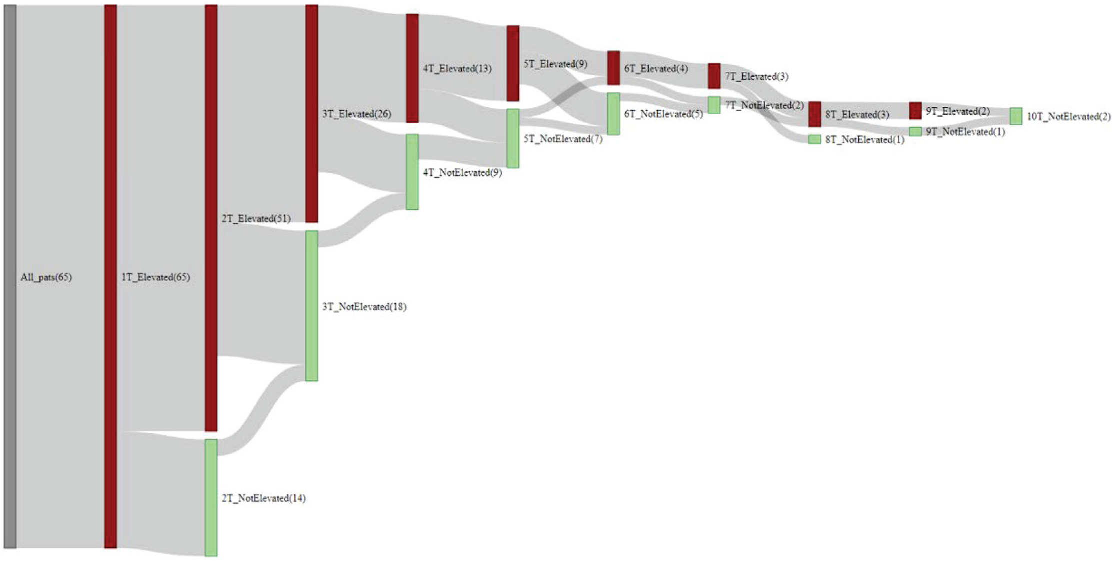

Figure 3. Sequence of AAV9-Ab titers for patients younger than 6 weeks old with elevated first test for AAV9-Ab who underwent repeat testing (n = 65)

access programs (median [range] age 4.8 [0.2–58.1] months) yielded 7.7% with elevated titers,[2] which is less than our findings. However, approximately 15.0% of mothers in this study had elevated titers,[2] which is consistent with the 17.9% of neonates in our study who had elevated titers, likely owing to transplacental maternal transfer. Interestingly, a recent study of 69 adults with SMA types 2 or 3 revealed that only 4.3% (n = 3) had elevated concentrations of AAV9-Ab, and the prevalence did not increase with age.[31] Adults with SMA are assumed to have a greater prevalence of AAV9-Ab, consistent with reports of AAV9-Ab in healthy adults (near 50.0%),[51] which would prohibit gene therapy for many patients. The reasons for the low prevalence and lack of age-related change reported in this study are unclear but may prompt consideration of further study of onasemnogene abeparvovec treatment for adults with SMA.

The prevalence of antibodies to AAV vectors differs according to serotype,[25,51,52] world[53,54] or US region,[55] and race,[56] but the reasons for those differences remain unclear. The geographic data collected here were not robust enough to confirm differences in the regional distribution of AAV9-Ab, possibly because of the low number of patients included in the analysis. Continued data collection will increase the amount of regional data available and allow for the discernment of clear geographic patterns in AAV9-Ab seroprevalence.

This analysis has several limitations. First, data were obtained from only two independent labs. Though tests have been validated internally by each lab, there is no consensus regarding assay standardization or definitive titers to determine elevation of AAV9-Ab. As described by Day et al., "Athena Diagnostics … defines patients with titers of <1:25 as being seronegative, whereas CTL and Viroclinics define patients with titers of ≤ 1:50 as being seronegative (i.e., in accordance with the criterion defined in the US Package Insert). The differences in these titers arise as a result of the different cutpoints used in the assays."[2] The differences in the sensitivity of the two tests did not allow for the direct comparison of titers, but the difference in these cutpoints formed the basis of the normalized titers. The lowest titers for which there was an elevated test result were considered E+1 (1:25 for Athena, 1:100 for CTL), whereas further elevations in the titers were considered E+2, E+3, and E+4. In addition, newborn screening for SMA was not routinely available during the entire study period. SMA screening was added to the Recommended Uniform Screening Panel by the FDA in 2018, and by 2021, only 34 states had fully implemented screening programs.[57] As of September 2022, 48 states had SMA screening programs.[58] In addition, care providers were able to test AAV9-Ab at their discretion, so testing times are inconsistent and may not accurately represent the time course of changes in AAV9-Ab concentrations. Finally, the study only included pediatric patients with SMA. Limited data are available to describe the worldwide seroprevalence of AAV9-Ab for adults with SMA, though at least one study reported a low prevalence (4.3%) of elevated titers for adults.[31] Titer concentrations in very young patients serve as an indirect measurement of maternal antibody titers, and our results may offer insight into the prevalence of elevated titers for women of child-bearing age in the United States who have children with SMA. The seroprevalence for pediatric patients without SMA has not been extensively studied. Future studies are needed to understand the seroprevalence of AAV9-Ab in larger and more general populations.

MATERIALS AND METHODS

We performed a retrospective secondary analysis of commercially available data for patients who underwent testing for antibodies that bind AAV9 as part of screening before treatment with onasemnogene abeparvovec. SMA patients who had commercially

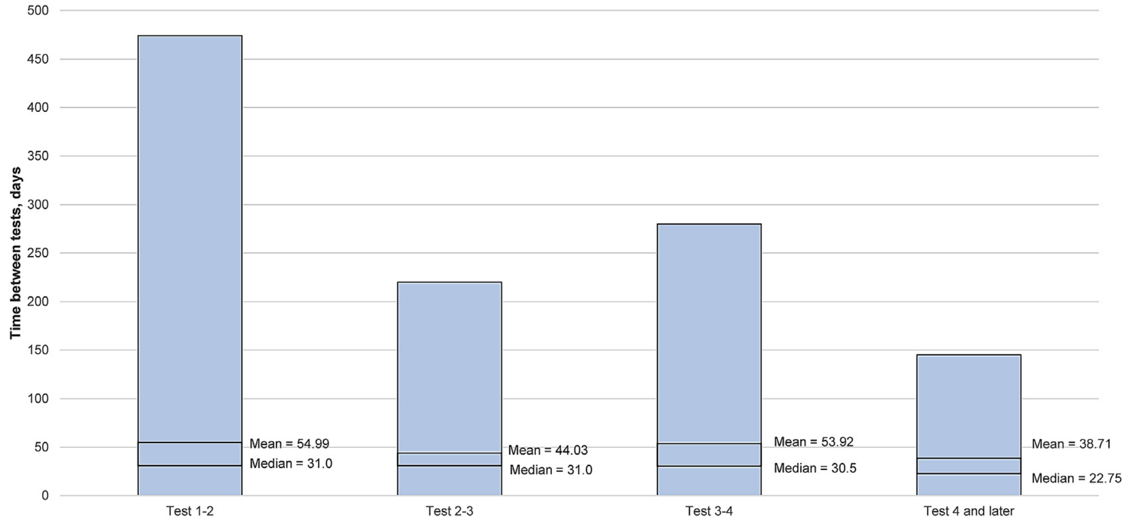

Figure 4. Time to retest for patients younger than 6 weeks old with elevated first test for AAV9-Ab who underwent repeat testing (n = 125)

available data and recorded consent for use of de-identified aggregated data were included. Patient data were excluded if there was no record of consent. Data were obtained from the United BioSource LLC (Blue Bell, PA, USA) US patient hub between February 15, 2019, and September 30, 2022. The dataset included patient characteristics (date of birth, *SMN2* copy number, sex), location (patient, health care provider, care system), AAV9-Ab testing (date, laboratory, final result), quantitative result (endpoint titers [last serum dilution in the standardized ELISA test using an indirect ELISA method]), and qualitative result (elevated vs. non-elevated). For the Athena test, a titer concentration ≥1:25 was considered elevated, and for the CTL test, >1:50 was considered elevated. The results were normalized for statistical analysis (Table S1), and the rationale for merging the data is explained in the discussion. No safety or efficacy data were obtained.

The primary objective was the evaluation of AAV9-Ab seroprevalence in patients in the United States with SMA. The secondary objectives were to identify regional variations in AAV9-Ab, to quantify the temporal decline in AAV9-Ab, to understand the time to first test for AAV9-Ab, and to understand the time to non-elevated AAV9-Ab to determine treatment eligibility.

The study was descriptive in nature, and no hypothesis was tested. Data analysis was completed using the latest version of R software (R Project, Boston, MA, USA). Results are reported as means (SDs), medians (ranges), or numbers (percentages).

CONCLUSIONS

For pediatric patients with SMA, elevated titers of AAV9-Ab at initial testing do not preclude treatment with onasemnogene abeparvovec gene therapy once titers decrease to normal levels. For young patients (<3 months old), lower magnitudes of elevated titers decline more rapidly—often within weeks—than greater magnitudes of elevated titers, which may take several months to decline to non-elevated levels. Overall, elevated AAV9-Ab titers are uncommon among infants and young children with SMA and almost all patients are eventually able to receive onasemnogene abeparvovec. Understanding the prevalence of preexisting AAV9-Ab is essential for evaluating the possibility of gene therapy for SMA.

DATA AND CODE AVAILABILITY

The data that support the findings of this study are available from United BioSource, LLC. Restrictions apply to the availability of these data, which were used under license for this study.

SUPPLEMENTAL INFORMATION

Supplemental information can be found online at https://doi.org/10.1016/j.omtm.2023.101117.

ACKNOWLEDGMENTS

Project management and documentation assistance was provided by Jamie Brading, Ciarán Pollard, and Priyesh Iyer. Data analysis was performed by Abhishek Srivastava, Shreya Banerjee, and Himanshu Karu from the CONEXTS-Real World Evidence team. Writing and editing assistance, including preparation of a draft manuscript under the direction and guidance of the authors, incorporating author feedback, and manuscript submission, was provided by Jennifer L. Gibson, PharmD (Kay Square Scientific, Newtown Square, PA, USA). This support was funded by Novartis Gene Therapies, Inc.

AUTHOR CONTRIBUTIONS

J.W.D., J.R.M., A.H.M.B., R.W.v.O., R.R.A., and K.W.D. performed the research and analyzed and interpreted the data. All authors contributed to the writing, editing, and review of the manuscript, and approved the submission of the manuscript.

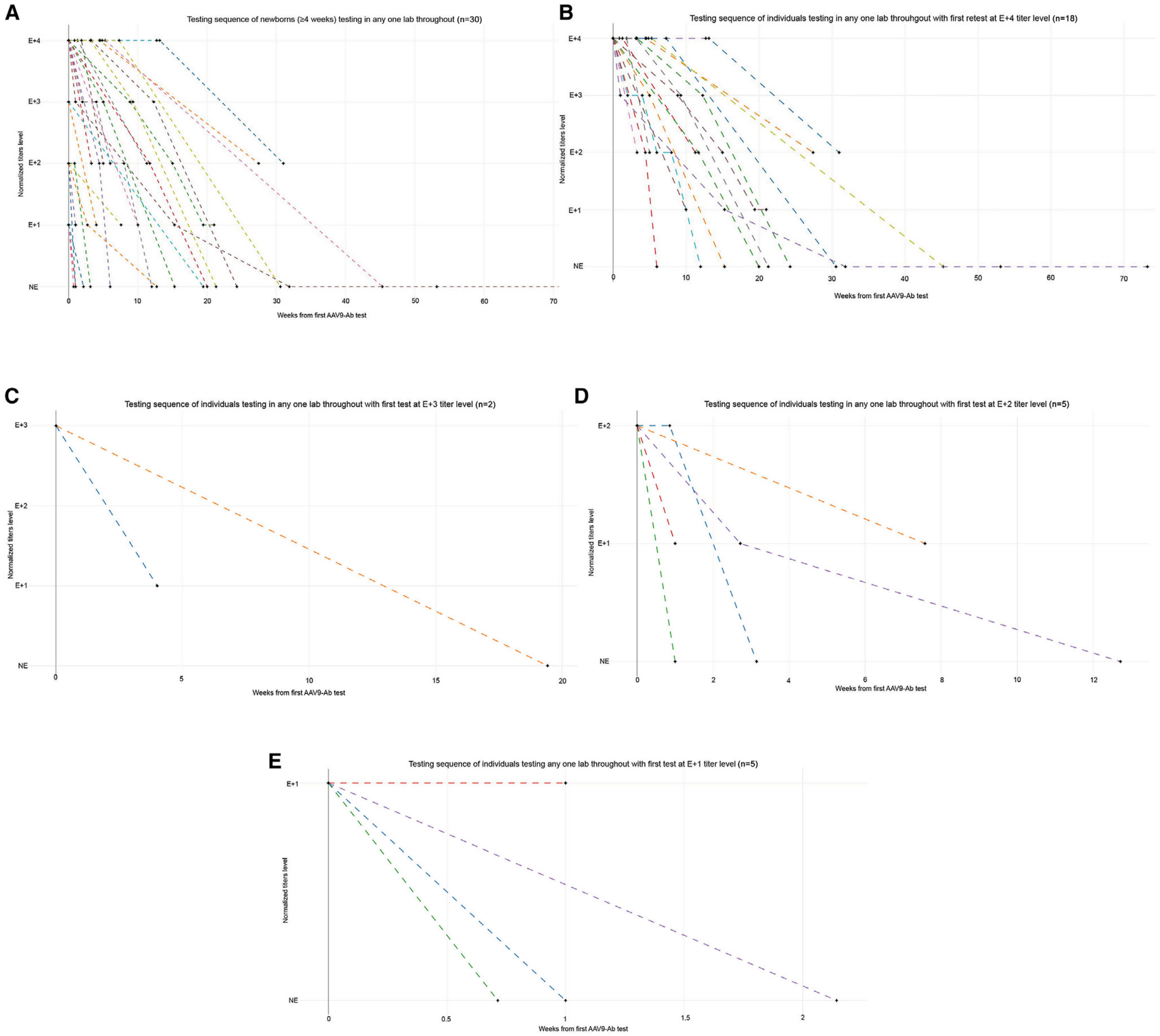

Figure 5. Testing sequence of newborns (≤4 weeks old) with repeat tests for AAV9-Ab performed at a single lab and with elevated first test result
(A) Testing sequence of newborns with an elevated first test (n = 30).
(B) Testing sequence of newborns with first test result at E+4 titer concentration (n = 18) (CTL: n = 3; Athena: n = 15).
(C) Testing sequence of newborns with first test result at E+3 titer concentration (n = 2) (Athena: n = 2).
(D) Testing sequence of newborns with first test result at E+2 titer concentration (n = 5) (CTL: n = 1; Athena: n = 4).
(E) Testing sequence of newborns with first test result at E+1 titer concentration (n = 5) (CTL: n = 1; Athena: n = 4). Note: Two patients had identical results and are represented by a single line. For the Athena test, a titer concentration ≥1:25 was considered elevated, and for the CTL test, >1:50 was considered elevated. AAV9-Ab, adeno-associated virus serotype 9 antibody; CTL, Cellular Technology Limited; E+1, 1:25 titer concentration on Athena test or 1:100 titer concentration on CTL test; E+2, 1:50 titer concentration on Athena test or 1:200 titer concentration on CTL test; E+3, 1:100 titer concentration on Athena test or 1:400 titer concentration on CTL test; E+4, ≥1:200 titer concentration on Athena test or ≥1:800 titer concentration on CTL test; NE, not elevated.

DECLARATION OF INTERESTS

J.W.D. has received consulting fees from Novartis Gene Therapies, Inc., Biogen, Cytokinetics, Ionis Pharmaceuticals, Pfizer, Roche, and Sarepta Therapeutics; license fees or royalty payments from Athena Diagnostics; and research funding from Novartis Gene Therapies, Inc., Biogen, Cytokinetics, Roche, Sanofi Genzyme, and Sarepta Therapeutics. J.R.M. has received personal compensation for clinical trial consulting, serving on scientific advisory boards, and research support from Novartis Gene Therapies, Inc. A.H.M.B. is a consultant for Novartis Gene Therapies, Inc., and conducted research for

Exicure, Inc. R.W.v.O. is an employee of Novartis Gene Therapies and owns Novartis stock or other equities. R.R.A. is an employee of Novartis Healthcare Private Limited, India. K.W.D. is an employee of Novartis Gene Therapies, Inc., and holds stock or other equities.

REFERENCES

1. Castle, M.J., Turunen, H.T., Vandenberghe, L.H., and Wolfe, J.H. (2016). Controlling AAV tropism in the nervous system with natural and engineering capsids. Methods Mol. Biol. *1382*, 133–149.
2. Day, J.W., Finkel, R.S., Mercuri, E., Swoboda, K.J., Menier, M., van Olden, R., Tauscher-Wisniewski, S., and Mendell, J.R. (2021). Adeno-associated virus serotype 9 antibodies in patients screened for treatment with onasemnogene abeparvovec. Mol. Ther. Methods Clin. Dev. *21*, 76–82.
3. DiMattia, M.A., Nam, H.J., Van Vliet, K., Mitchell, M., Bennett, A., Gurda, B.L., McKenna, R., Olson, N.H., Sinkovits, R.S., Potter, M., et al. (2012). Structural insight into the unique properties of adeno-associated virus serotype 9. J. Virol. *86*, 6947–6958.
4. Al-Zaidy, S.A., and Mendell, J.R. (2019). From clinical trials to clinical practice: practical considerations for gene replacement therapy in SMA type 1. Pediatr. Neurol. *100*, 3–11.
5. Jablonka, S., Hennlein, L., and Sendtner, M. (2022). Therapy development for spinal muscular atrophy: perspectives for muscular dystrophies and neurodegenerative disorders. Neurol. Res. Pract. *11*, 31.
6. Kotulska, K., Fattal-Valevski, A., and Haberlova, J. (2021). Recombinant adeno-associated virus serotype 9 gene therapy in spinal muscular atrophy. Front. Neurol. *12*, 726468.
7. Lefebvre, S., Bürglen, L., Reboullet, S., Clermont, O., Burlet, P., Viollet, L., Benichou, B., Cruaud, C., Millasseau, P., and Zeviani, M. (1995). Identification and characterization of a spinal muscular atrophy-determining gene. Cell *80*, 155–165.
8. Coovert, D.D., Le, T.T., McAndrew, P.E., Strasswimmer, J., Crawford, T.O., Mendell, J.R., Coulson, S.E., Androphy, E.J., Prior, T.W., and Burghes, A.H. (1997). The survival motor neuron protein in spinal muscular atrophy. Hum. Mol. Genet. *6*, 1205–1214.
9. Lefebvre, S., Burlet, P., Liu, Q., Bertrandy, S., Clermont, O., Munnich, A., Dreyfuss, G., and Melki, J. (1997). Correlation between severity and SMN protein level in spinal muscular atrophy. Nat. Genet. *16*, 265–269.
10. Bladen, C.L., Thompson, R., Jackson, J.M., Garland, C., Wegel, C., Ambrosini, A., Pisano, P., Walter, M.C., Schreiber, O., Lusakowska, A., et al. (2014). Mapping the differences in care for 5,000 spinal muscular atrophy patients, a survey of 24 national registries in North America, Australasia and Europe. J. Neurol. *261*, 152–163.
11. Kolb, S.J., Coffey, C.S., Yankey, J.W., Krosschell, K., Arnold, W.D., Rutkove, S.B., Swoboda, K.J., Reyna, S.P., Sakonju, A., Darras, B.T., et al. (2017). Natural history of infantile-onset spinal muscular atrophy. Ann. Neurol. *82*, 883–891.
12. Duque, S.I., Arnold, W.D., Odermatt, P., Li, X., Porensky, P.N., Schmelzer, L., Meyer, K., Kolb, S.J., Schümperli, D., Kaspar, B.K., and Burghes, A.H.M. (2015). A large animal model of spinal muscular atrophy and correction of phenotype. Ann. Neurol. *77*, 399–414.
13. Thomsen, G., Burghes, A.H.M., Hsieh, C., Do, J., Chu, B.T.T., Perry, S., Barkho, B., Kaufmann, P., Sproule, D.M., Feltner, D.E., et al. (2021). Biodistribution of onasemnogene abeparvovec DNA, mRNA and SMN protein in human tissue. Nat. Med. *27*, 1701–1711.
14. Day, J.W., Finkel, R.S., Chiriboga, C.A., Connolly, A.M., Crawford, T.O., Darras, B.T., Iannaccone, S.T., Kuntz, N.L., Peña, L.D.M., Shieh, P.B., et al. (2021). Onasemnogene abeparvovec gene therapy for symptomatic infantile-onset spinal muscular atrophy in patients with two copies of SMN2 (STR1VE): an open-label, single-arm, multicentre, phase 3 trial. Lancet Neurol. *20*, 284–293.
15. Mendell, J.R., Al-Zaidy, S., Shell, R., Arnold, W.D., Rodino-Klapac, L.R., Prior, T.W., Lowes, L., Alfano, L., Berry, K., Church, K., et al. (2017). Single-dose gene-replacement therapy for spinal muscular atrophy. N. Engl. J. Med. *377*, 1713–1722.
16. Mendell, J.R., Al-Zaidy, S.A., Lehman, K.J., McColly, M., Lowes, L.P., Alfano, L.N., Reash, N.F., Iammarino, M.A., Church, K.R., Kleyn, A., et al. (2021). Five-year extension results of the phase 1 START trial of onasemnogene abeparvovec in spinal muscular atrophy. JAMA Neurol. *78*, 834–841.
17. Mercuri, E., Muntoni, F., Baranello, G., Masson, R., Boespflug-Tanguy, O., Bruno, C., Corti, S., Daron, A., Deconinck, N., Servais, L., et al. (2021). Onasemnogene abeparvovec gene therapy for symptomatic infantile-onset spinal muscular atrophy type 1 (STR1VE-EU): an open-label, single-arm, multicentre, phase 3 trial. Lancet Neurol. *20*, 832–841.
18. Strauss, K.A., Farrar, M.A., Muntoni, F., Saito, K., Mendell, J.R., Servais, L., McMillan, H.J., Finkel, R.S., Swoboda, K.J., Kwon, J.M., et al. (2022). Onasemnogene abeparvovec for presymptomatic infants with three copies of SMN2 at risk for spinal muscular atrophy: the phase 3 SPR1NT trial. Nat. Med. *28*, 1390–1397.
19. Strauss, K.A., Farrar, M.A., Muntoni, F., Saito, K., Mendell, J.R., Servais, L., McMillan, H.J., Finkel, R.S., Swoboda, K.J., Kwon, J.M., et al. (2022). Onasemnogene abeparvovec for presymptomatic infants with two copies of SMN2 at risk for spinal muscular atrophy type 1: the phase 3 SPR1NT trial. Nat. Med. *28*, 1381–1389.
20. Colella, P., Ronzitti, G., and Mingozzi, F. (2018). Emerging issues in AAV-mediated *in vivo* gene therapy. Mol. Ther. Methods Clin. Dev. *8*, 87–104.
21. Smith, R.H. (2008). Adeno-associated virus integration: virus versus vector. Gene Ther. *15*, 817–822.
22. Albrecht, M., Pagenkemper, M., Wiessner, C., Spohn, M., Lütgehetmann, M., Jacobsen, H., Gabriel, G., Zazara, D.E., Haertel, C., Hecher, K., et al. (2022). Infant immunity against viral infections is advanced by the placenta-dependent vertical transfer of maternal antibodies. Vaccine *40*, 1563–1571.
23. Pou, C., Nkulikiyimfura, D., Henckel, E., Olin, A., Lakshmikanth, T., Mikes, J., Wang, J., Chen, Y., Bernhardsson, A.K., Gustafsson, A., et al. (2019). The repertoire of maternal anti-viral antibodies in human newborns. Nat. Med. *25*, 591–596.
24. Van de Perre, P. (2003). Transfer of antibody via mother's milk. Vaccine *21*, 3374–3376.
25. Calcedo, R., Morizono, H., Wang, L., McCarter, R., He, J., Jones, D., Batshaw, M.L., and Wilson, J.M. (2011). Adeno-associated virus antibody profiles in newborns, children, and adolescents. Clin. Vaccine Immunol. *18*, 1586–1588.
26. Calcedo, R., and Wilson, J.M. (2013). Humoral immune response to AAV. Front. Immunol. *4*, 341.
27. Verdera, H.C., Kuranda, K., and Mingozzi, F. (2020). AAV vector immunogenicity in humans: a long journey to successful gene transfer. Mol. Ther. *28*, 723–746.
28. Erles, K., Sebökovà, P., and Schlehofer, J.R. (1999). Update on the prevalence of serum antibodies (IgG and IgM) to adeno-associated virus (AAV). J. Med. Virol. *59*, 406–411.
29. Fu, H., Meadows, A.S., Pineda, R.J., Kunkler, K.L., Truxal, K.V., McBride, K.L., Flanigan, K.M., and McCarty, D.M. (2017). Differential prevalence of antibodies against adeno-associated virus in healthy children and parents with mucopolysaccharidosis III: perspective for AAV-mediated gene therapy. Hum. Gene Ther. Clin. Dev. *28*, 187–196.
30. Li, C., Narkbunnam, N., Samulski, R.J., Asokan, A., Hu, G., Jacobson, L.J., Manco-Johnson, M.J., and Monahan, P.E. (2012). Joint Outcome Study Investigators. Neutralizing antibodies against adeno-associated virus examined prospectively in pediatric patients with hemophilia. Gene Ther *19*, 288–294.
31. Stolte, B., Schreiber-Katz, O., Günther, R., Wurster, C.D., Petri, S., Osmanovic, A., Freigang, M., Uzelac, Z., Leo, M., von Velsen, O., et al. (2022). Prevalence of anti-AAV9 antibodies in adult patients with spinal muscular atrophy. Hum. Gene Ther. *33*, 968–976.
32. Wang, D., Tai, P.W.L., and Gao, G. (2019). Adeno-associated virus vector as a platform for gene therapy delivery. Nat. Rev. Drug Discov. *18*, 358–378.
33. Barnes, C., Scheideler, O., and Schaffer, D. (2019). Engineering the AAV capsid to evade immune responses. Curr. Opin. Biotechnol. *60*, 99–103.
34. Büning, H., and Srivastava, A. (2019). Capsid modifications for targeting and improving the efficacy of AAV vectors. Mol. Ther. Methods Clin. Dev. *12*, 248–265.
35. Gray, S.J., Matagne, V., Bachaboina, L., Yadav, S., Ojeda, S.R., and Samulski, R.J. (2011). Preclinical differences of intravascular AAV9 delivery to neurons and glia: a comparative study of adult mice and nonhuman primates. Mol. Ther. *19*, 1058–1069.

36. Zolgensma [onasemnogene abeparvovec-xioi] (2021). Prescribing Information (Novartis Gene Therapies, Inc).

37. Mendell, J.R., Sahenk, Z., Lehman, K., Nease, C., Lowes, L.P., Miller, N.F., Iammarino, M.A., Alfano, L.N., Nicholl, A., Al-Zaidy, S., et al. (2020). Assessment of systemic delivery of rAAVrh74.MHCK7.micro-dystrophin in children with Duchenne muscular dystrophy: A nonrandomized controlled trial. JAMA Neurol. *77*, 1122–1131.

38. Kichula, E.A., Proud, C.M., Farrar, M.A., Kwon, J.M., Saito, K., Desguerre, I., and McMillan, H.J. (2021). Expert recommendations and clinical considerations in the use of onasemnogene abeparvovec gene therapy for spinal muscular atrophy. Muscle Nerve *64*, 413–427.

39. Ballreich, J., Ezebilo, I., Khalifa, B.A., Choe, J., and Anderson, G. (2022). Coverage of genetic therapies for spinal muscular atrophy across fee-for-service Medicaid programs. J. Manag. Care Spec. Pharm. *28*, 39–47.

40. Lee, B.H., Waldrop, M.A., Connolly, A.M., and Ciafaloni, E. (2021). Time is muscle: A recommendation for early treatment for preterm infants with spinal muscular atrophy. Muscle Nerve *64*, 153–155.

41. Bashiri, F.A., Temsah, M.-H., Hundallah, K., Alsohime, F., and AlRuthia, Y. (2021). 2020 Update to spinal muscular atrophy management in Saudi Arabia. Front. Pediatr. *9*, 684134.

42. Ramdas, S., and Servais, L. (2020). New treatments in spinal muscular atrophy: an overview of currently available data. Expert Opin. Pharmacother. *21*, 307–315.

43. Mercuri, E., Finkel, R.S., Muntoni, F., Wirth, B., Montes, J., Main, M., Mazzone, E.S., Vitale, M., Snyder, B., Quijano-Roy, S., et al. (2018). Diagnosis and management of spinal muscular atrophy: Part 1: recommendations for diagnosis, rehabilitation, orthopedic and nutritional care. Neuromuscul. Disord. *28*, 103–115.

44. Finkel, R.S., Mercuri, E., Meyer, O.H., Simonds, A.K., Schroth, M.K., Graham, R.J., Kirschner, J., Iannaccone, S.T., Crawford, T.O., Woods, S., et al. (2018). Diagnosis and management of spinal muscular atrophy: Part 2: pulmonary and acute care; medications, supplements, and immunizations; other organ systems; and ethics. Neuromuscul. Disord. *28*, 197–207.

45. Chaytow, H., Faller, K.M.E., Huang, Y.T., and Gillingwater, T.H. (2021). Spinal muscular atrophy: from approved therapies to future therapeutic targets for personalized medicine. Cell Rep. Med. *2*, 100346.

46. Chen, T.H. (2020). New and developing therapies in spinal muscular atrophy: from genotype to phenotype to treatment and where do we stand? Int. J. Mol. Sci. *21*, 3297.

47. Lee, B.H., Collins, E., Lewis, L., Guntrum, D., Eichinger, K., Voter, K., Abdel-Hamid, H.Z., and Ciafaloni, E. (2019). Combination therapy with nusinersen and AVXS-101 in SMA type 1. Neurology *93*, 640–641.

48. Kray, K.M., McGovern, V.L., Chugh, D., Arnold, W.D., and Burghes, A.H.M. (2021). Dual SMN inducing therapies can rescue survival and motor function in symptomatic Δ7SMA mice. Neurobiol. Dis. *159*, 105488.

49. Ferrante, L., Melendez-Zaidi, A., Lindsey, W., and Lotze, T. (2022). Novel use of nusinersen as a therapeutic bridge to onasemnogene abeparvovec-xioi in a premature neonate with type 1 spinal muscular atrophy. Muscle Nerve *66*, E8–E10.

50. Aharoni, S., Bistritzer, J., Levine, H., Sagi, L., Fattal-Valevski, A., Ginzberg, M., Noyman, I., Cohen, R., and Nevo, Y. (2023). Adeno-associated virus serotype 9 antibody titers in patients with SMA pre-screened for treatment with onasemnogene abeparvovec – routine care evidence. Gene Ther. *30*, 101–106.

51. Boutin, S., Monteilhet, V., Veron, P., Leborgne, C., Benveniste, O., Montus, M.F., and Masurier, C. (2010). Prevalence of serum IgG and neutralizing factors against adenoassociated virus (AAV) types 1,2, 5, 6, 8, and 9 in the healthy population: implications for gene therapy using AAV vectors. Hum. Gene Ther. *21*, 704–712.

52. Vandamme, C., Adjali, O., and Mingozzi, F. (2017). Unraveling the complex story of immune responses to AAV vectors trial after trial. Hum. Gene Ther. *28*, 1061–1074.

53. Calcedo, R., Vandenberghe, L.H., Gao, G., Lin, J., and Wilson, J.M. (2009). Worldwide epidemiology of neutralizing antibodies to adeno-associated viruses. J. Infect. Dis. *199*, 381–390.

54. Kruzik, A., Fetahagic, D., Hartlieb, B., Dorn, S., Koppensteiner, H., Horling, F.M., Scheiflinger, F., Reipert, B.M., and de la Rosa, M. (2019). Prevalence of anti-adeno-associated virus immune responses in international cohorts of healthy donors. Mol. Ther. Methods Clin. Dev. *14*, 126–133.

55. Cao, L., Ledeboer, A., Pan, Y., Lu, Y., and Meyer, K. (2023). Clinical enrollment assay to detect preexisting neutralizing antibodies to AAV6 with demonstrated transgene expression in gene therapy trials. Gene Ther. *30*, 150–159.

56. Khatri, A., Shelke, R., Guan, S., and Somanathan, S. (2022). Higher seroprevalence of anti-adeno-associated viral vector neutralizing antibodies among racial minorities in the United States. Hum. Gene Ther. *33*, 442–450.

57. Hale, K., Ojodu, J., and Singh, S. (2021). Landscape of spinal muscular atrophy newborn screening in the United States: 2018–2021. Int. J. Neonatal Screen. *7*, 33.

58. CureSMA. Newborn screening for SMA. https://www.curesma.org/newborn-screening-for-sma/.

OMTM, Volume *31*

Supplemental information

Adeno-associated virus serotype 9 antibody seroprevalence for patients in the United States with spinal muscular atrophy

John W. Day, Jerry R. Mendell, Arthur H.M. Burghes, Rudolf W. van Olden, Rishi R. Adhikary, and Keith W. Dilly

Table S1. Reference table for normalized titers

Lab	Technical results	Elevated/NE	Normalized titers
Athena	<1:25	NE	NE
Athena	1:25	Elevated	E+1
Athena	1:50	Elevated	E+2
Athena	1:100	Elevated	E+3
Athena	≥1:200	Elevated	E+4
CTL	<1:50	NE	NE
CTL	1:50	NE	NE
CTL	1:100	Elevated	E+1
CTL	1:200	Elevated	E+2
CTL	1:400	Elevated	E+3
CTL	1:800	Elevated	E+4
CTL	>1:800 & 1:1600	Elevated	E+4

CTL, Cellular Technology Limited; NE, not elevated. Note: For the Athena test, a titer concentration ≥1:25 was considered elevated, and for the CTL test, >1:50 was considered elevated.

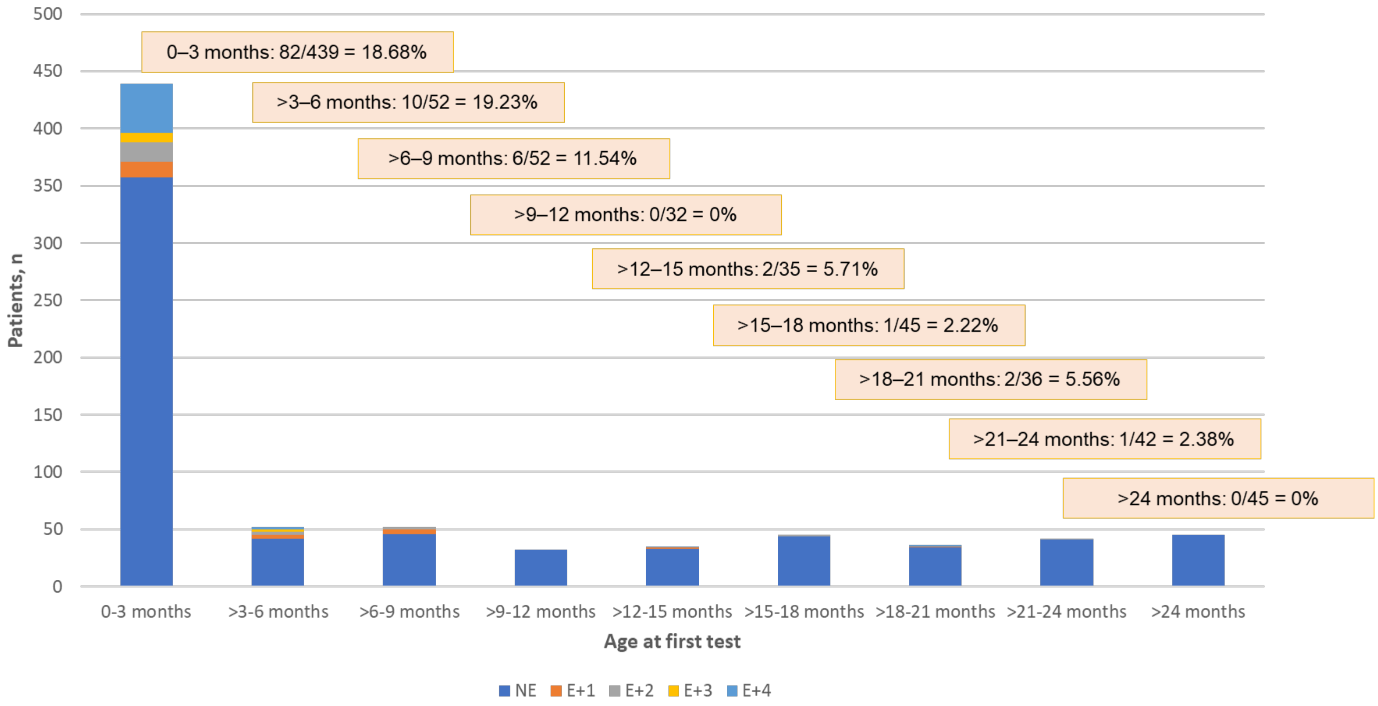

a) Patients with first test completed by Athena Diagnostics (n=778)

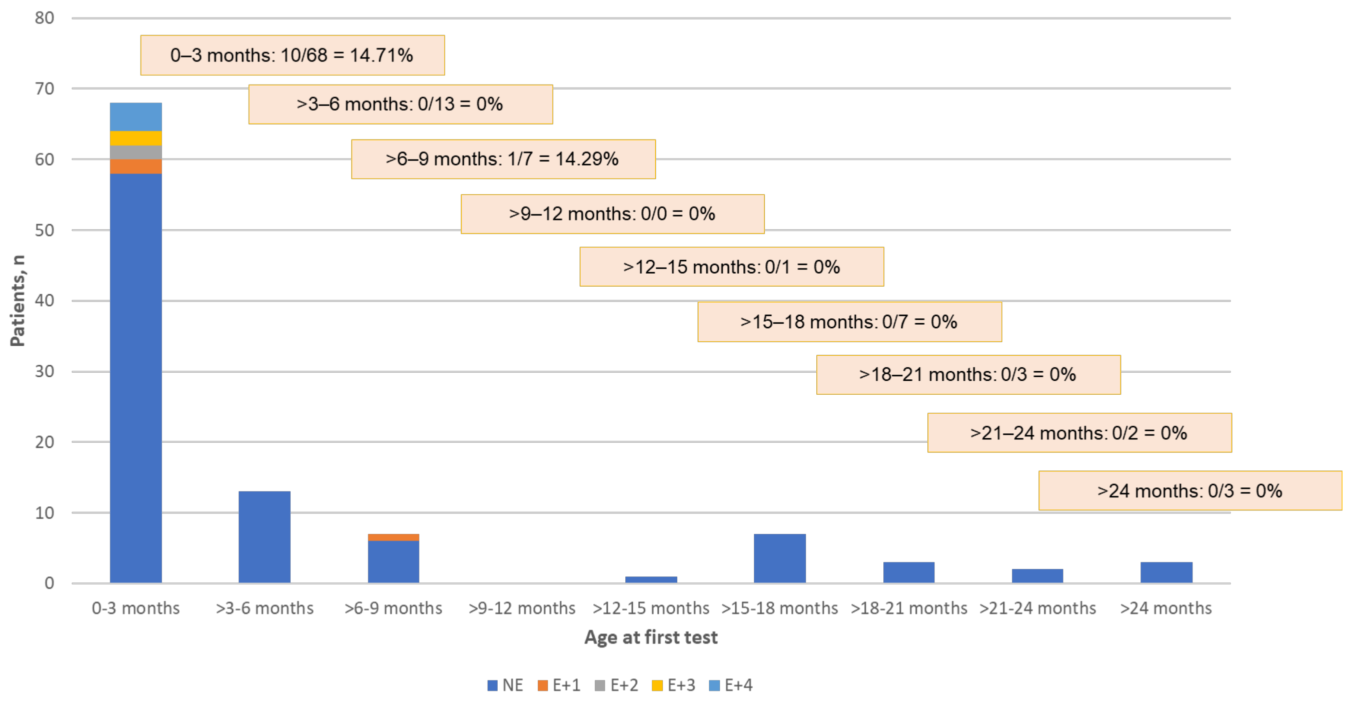

b) Patients with first test completed by CTL (n=104)

Figure S1. Age distribution of normalized titers at first test for antibodies that bind AAV9 according to laboratory that conducted the test.

CTL, Cellular Technology Limited; E+1, 1:25 titer concentration on Athena test or 1:100 titer concentration on CTL test; E+2, 1:50 titer concentration on Athena test or 1:200 titer concentration on CTL test; E+3, 1:100 titer concentration on Athena test or 1:400 titer concentration on CTL test; E+4, ≥1:200 titer concentration on Athena test or ≥1:800 titer concentration on CTL test; NE, not elevated. Note: For the Athena test, a titer concentration ≥1:25 was considered elevated, and for the CTL test, >1:50 was considered elevated.

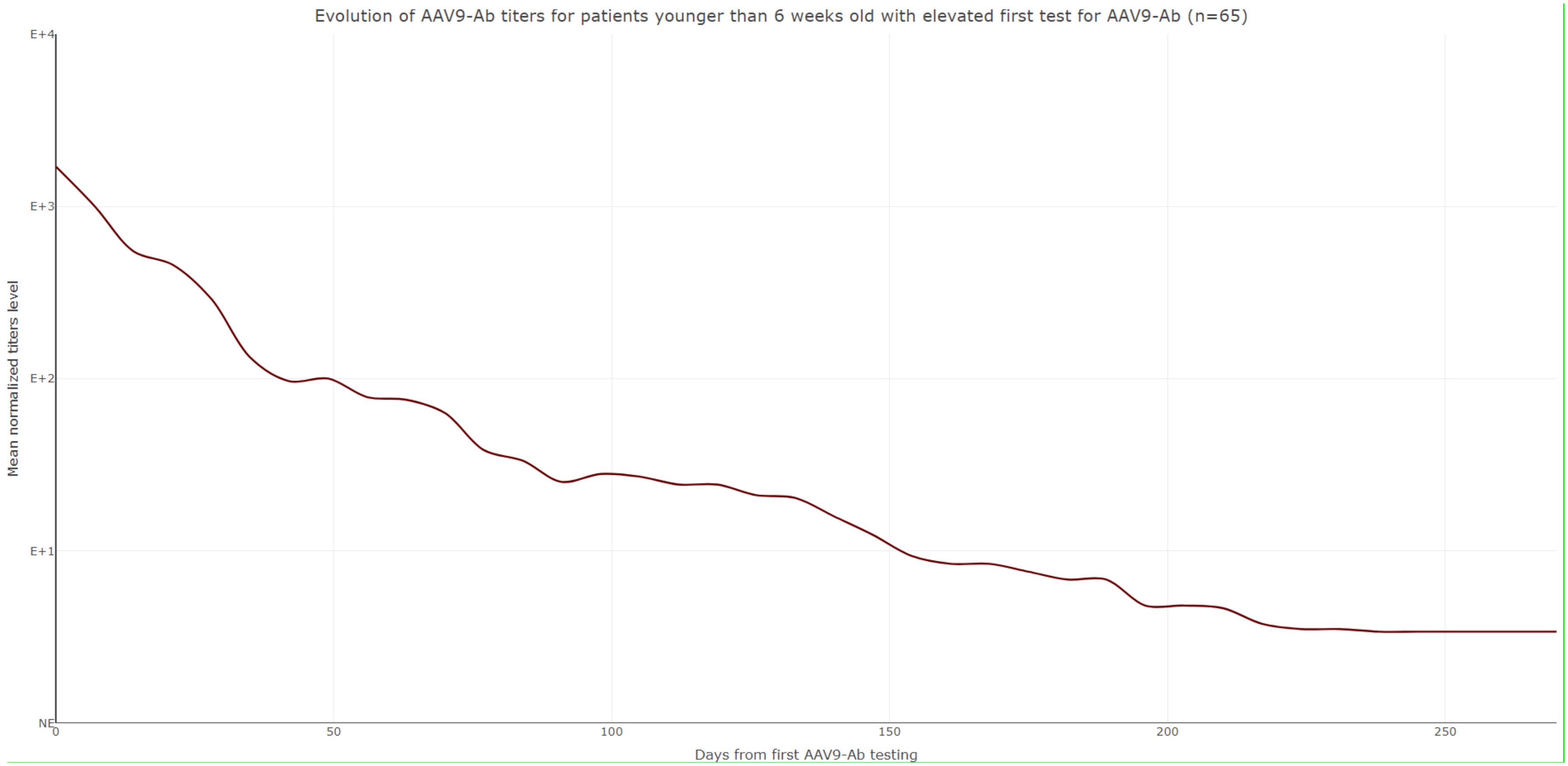

Figure S2. Evolution of AAV9-Ab titers for patients younger than 6 weeks old with elevated first test for AAV9-Ab who underwent repeat testing (n=65)

AAV9-Ab, adeno-associated virus serotype 9 antibodies.
